# Gait Recognition by Combining the Long-Short-Term Attention Network and Personal Physiological Features

**DOI:** 10.3390/s22228779

**Published:** 2022-11-14

**Authors:** Chunsheng Hua, Yingjie Pan, Jia Li, Zhibo Wang

**Affiliations:** 1 Institute of Intelligent Robot and Pattern Recognition, College of Information, Liaoning University, No. 66 Chongshan Middle Road, Huanggu District, Shenyang 110036, China; 2 College of Information, Liaoning University, Shenyang 110036, China; 3 Department of Endocrinology and Metabolism, The Fourth Affiliated Hospital of China Medical University, Shenyang 110096, China; 4 Shenyang Contain Electronic Technology Co., Ltd., Shenyang 110167, China

**Keywords:** gait recognition, biometrics, feature extraction, feature fusion, image sequence, deep learning

## Abstract

Although gait recognition has been greatly improved by efforts from many researchers in recent years, its performance is still unsatisfactory due to the lack of gait information under the real scenariowhere only one or two images may be used for recognition. In this paper, a new gait recognition framework is brought about which can combine the long-short-term attention modules on silhouette images over the whole sequence and the real human physiological information calculated by a monocular image. The contributions of this work include the following: (1) Fusing the global long-term attention (GLTA) and local short-term attention (LSTA) over the whole query sequence to improve the gait recognition accuracy, where both the short-term gait feature (from two or three frames) and long-term feature (from the whole sequence) are extracted; (2) presenting a method to calculate the real personal static and dynamic physiological features through a single monocular image; (3) by efficiently applying the human physiological information, a new physiological feature extraction (PFE) network is proposed to concatenate the physiological information with silhouette for gait recognition. Through the experiments between the CASIA-B and Multi-state Gait datasets, the effectiveness and efficiency of the proposed method are proven. Under three different walking conditions of the CASIA-B dataset, the mean accuracy of rank-1 in our method is up to 89.6%, and in the Multi-state Gait dataset, wearing different clothes, the mean accuracy of rank-1 in our method is 2.4% higher than the other works.

## 1. Introduction

Gait recognition has drawn attention from numerous researchers as an important biometric recognition algorithm. It plays an import role in many tasks, such as surveillance systems, anti-terrorist operations, clinic diagnosis, etc. Unlike some biometric features that need to be extracted at close range (such as face or iris) or by touch-sensor (fingerprint), gait features can be effectively collected even if the target person is at a distance of 20 m from the camera. The extraction of gait features does not need the intentional cooperation of the target person, and can be widely applied in surveillance videos, biometric measurement, dialog, etc. Meanwhile, gait features, as habitual human movement features, will not be easily changed. However, there are still many challenges among the conventional gait recognition algorithms; this is because the extraction of gait features can be affected by different factors, such as the variation of camera view angles and different walking directions of pedestrians. Some works [1,2,3] have shown that in real scenes, the changes in clothing, object occlusion, and pedestrian walking speed can also affect the feature extraction.

In recent years, some methods have been proven to be very effective in extracting gait features, which could be categorized in two ways: the discriminative model-based algorithms [4,5,6,7,8,9,10,11] and the generative model-based ones [12,13]. Regarding the discriminative model-based algorithms, the identity discrimination is mainly performed by extracting features in gait templates or gait sequences. The gait energy image [14] is a gait template generated from the gait silhouette, which is obtained by time-averagedpooling. In [4], Shiraga et al. used gait energy images for feature representation to obtain the perspective invariant features through fully connected layers. Wu et al. [8] selected deep convolutional neural networks to learn the similarity between gait energy images for identity discrimination. Although the gait energy image can reduce the computational cost, it will lose the frame level features. Therefore, in recent years, researchers have tended to extract features directly from the frame sequence [5,6]. Since neither a single spatial nor a temporal feature can provide complete access to the information, neither of them could provide enough information for gait recognition alone. Chao et al. [5] considered that a gait sequence is composed of both spatial location and temporal information, and took the gait sequence as an unordered set for feature extraction. Fan et al. [6] proposed a focal convolutional layer to refine the feature extraction, and brought out a novel local gait feature representation to describe the spatiotemporal features of the human body in a short period of time, where such features have been proven to be superior to other ones. Inspired by [15] who proposedthat 3D CNN could efficiently extract both the spatial features and temporal ones, Wolf et al. [9] applied 3D CNN to extract the spatiotemporal information in gait sequences in order to solve the problems caused by the indefinite length of gait sequence, where the gait sequences were cut into several short ones. In [10], Thapar et al. used 3D CNN for feature extraction from different viewpoints. On the other hand, in [11], Liao used the extracted human key point information for gait recognition, and added three posture features: joint angle, limb length, and joint motion. Liao et al. [7] used human pose information as the input of the model, and mentioned that RNN [16] or LSTM [17] are used to extract time information from a sequence. Regarding the generative model-based algorithms, the operation of encoding and decoding the gait sequence features is required; Feng et al. [12] applied the LSTM to process the obtained nodes and reconstruct the gait sequence features from different viewpoints, while Yu et al. [13] used generative adversarial networks to reduce the effects caused by clothing changes, viewpoint changes, etc. Although the silhouette image of a person may change greatly (due to the variation of view angles among the surveillance cameras), it is well known that a person’s real physiological information (such as his real height, shoulder width, step frequency, and other information) will not actually change greatly. In [18,19], such physiological information was reported to be able to improve the accuracy of gait recognition algorithms.

In this paper, a novel gait recognition framework is brought about with the combination of long-short-term global/local features and real personal physiological information. Since the movement frequency of persons may change greatly, both the local short-term attention (LSTA, for three continuous frames) and global long-term attention (GLTA, for the whole gait cycle) modules are proposed to collect more effective gait features. Based on the observation that a silhouette image can only provide limited shape or motion features, a novel human physiological information (HPI) module is also brought about for calculating the real personal static and dynamic physiological features through the monocular images. To efficiently apply the HPI features, a new physiological feature extraction (PFE) network is proposed to concatenate the physiological information with silhouette for gait recognition. Through the experiments between the CASIA-B [20] and Multi-state Gait (collected by us) datasets, the effectiveness and efficiency of the proposed method are proved.

## 2. Methods

### 2.1. System Overview

**Figure 1:**
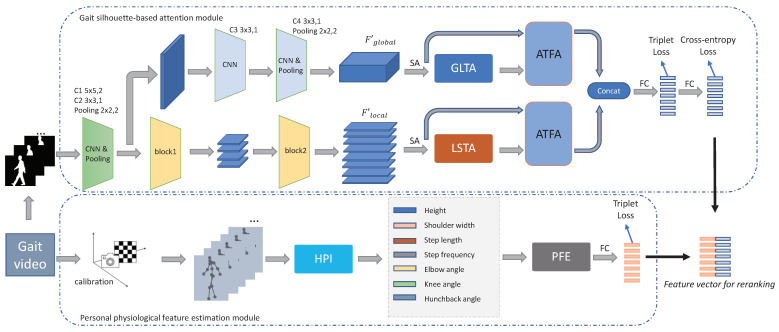
The overviewof this work, which is mainly composed of two parts: the gait-silhouette-based global (local) long (short)-term attention module, and the personal physiological feature estimation module.

The main structure of this paper is shown in Figure 1, which mainly includes two parts: the gait-silhouette-based attention module and real personal physiological feature estimation module.

In the gait-silhouette-based attention module, the local short-term attention (LSTA) features and global long-term attention (GLTA) features are extracted by the different multilayer perceptronand feature aggregation. Then, the feature aggregation is performed by using multiple features to complement each other. Regarding the global long-term features, four layers of 2D CNN and two layers of max pooling are used to obtain the shallow feature information, and then the global long-term features of the whole sequence are extracted using the global long-term attention module (GLTA). Finally, the features are aggregated by the adaptive temporal feature aggregation module (ATFA). Regarding the local short-term features, GaitPart [6] is selected as the backbone network to extract shallow features, and then the local short-term features are extracted using the local short-term attention module (LSTA), where the extracted features are also aggregated by the ATFA module.

During the personal physiological feature estimation module, after the camera calibration, the personal physiological information (such as shoulder width, step length, frequency, etc.) is extracted from the skeletal points through the input monocular images by the human physiological information module (HPI). Then, the physiological feature extraction module (PFE) is used to extract and aggregate each of the gait physiological features. The full connectivity (FC) layers are used to map the feature vector into the metric space, and the features obtained from the two modules are concatenated for reranking.

### 2.2. Gait-Silhouette-Based Attention Modules

A similar local convolution network to GaitPart [6] is brought about to extract local features of different receptive fields. As shown in Figure 1, block1 and block2 are applied to split the input feature map into four and eight parts horizontally by focal convolution layers [6]. Here, the local feature dimension is represented by ${{F^{\prime }}_{local}\in R}^{N\times P\times S\times C\times (H/P)\times W}$, where *N* is the batch, *S* means the time series, *C* represents the feature channel, *H* and *W* are the height and width of the feature map, and *P* denotes the number of timesthe feature map is split. The global features are extracted by a four-layer 2D CNN, and represented as ${{F^{\prime }}_{global}\in R}^{N\times S\times C\times H\times W}$. Spatial aggregation (*SA*) refers to horizontal feature aggregation on the width *W* dimension of an image. The *SA* operations are described in Equations (1) and (2) to obtain the local features ${F_{local}\in R}^{N\times P\times S\times C\times (H/P)}$ and global features ${F_{global}\in R}^{N\times S\times C\times H}$, respectively. The ${\mathrm{avg}}_{W}$ and $\max_{W}$ mean the average and maximum value in width *W*.

(1) $${F_{local}\in R}^{N\times P\times S\times C\times (H/P)}=\;{\mathrm{avg}}_{W}\;\left({F^{\prime }}_{local}\right)+\max_{W}\;\left({F^{\prime }}_{local}\right),$$

(2) $${F_{global}\in R}^{N\times S\times C\times H}=\;{\mathrm{avg}}_{W}\;\left({F^{\prime }}_{global}\right)+\max_{W}\;\left({F^{\prime }}_{global}\right).$$

#### 2.2.1. Local Short-Term Attention (LSTA)

**Figure 2:**
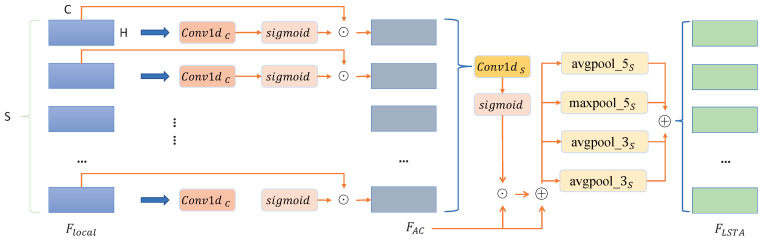
The overview structure of the LSTA.

As shown in Figure 2, a channel attention module is introduced to enhance the feature representation of the local features, A channel-based attention feature ${F_{AC}\in R}^{N\times P\times S\times C\times (H/P)}$ is described in Equation (3), where its distribution is obtained by using 1D CNN and a sigmoid function on the channel *C*. In Equation (3), a channel attention element-wise product over local features $F_{local}$ is selected to obtain the channel excitation features, $F_{AC}$.

(3) $$F_{AC}=Sigmoid\left({Conv1d\;}_{C}\left(F_{local}\right)\right)\cdot \;F_{local}.$$

After that, a one-dimensional convolution of size 1 is used in the time dimension to obtain temporal attention features ${F_{AS}\in R}^{N\times P\times S\times C\times (H/P)}$ of each row. Then, the average and max pooling with the sizes of 3 and 5 are used to slide into the time series *S* so as to extract the short-term features of different receptive fields, which can be defined as Equations (4) and (5), to obtain local short-term attention features ${F_{LSTA}\in R}^{N\times S\times C\times H}$ based on time series.

(4) $$F_{LSTA}={\mathrm{maxpool}\_3}_{S}\left(F_{AS}\right)\;+\;{\mathrm{avgpool}\_3}_{S}\left(F_{AS}\right)+{\mathrm{maxpool}\_5}_{S}\left(F_{AS}\right)+\;{\mathrm{avgpool}\_5}_{S}\left(F_{AS}\right),$$

(5) $$F_{AS}=Sigmoid\left({Conv1d\;}_{S}\left(F_{AC}\right)\right)•F_{AC}+F_{AC}.$$

#### 2.2.2. Global Long-Term Attention (GLTA)

**Figure 3:**
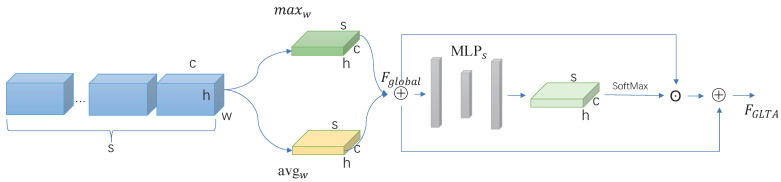
The overview of GLTA.

Besides the local features, the global features could also be useful to describe the holistic information of the target person. As described in Figure 3, after obtaining a gait silhouette sequence of *S* frames, the shallow features $F_{global}$ of the whole sequence are obtained by the *SA* operation (described in Equations (1) and (2)). In order to extract a more discriminative feature representation, the importance of each frame in the whole sequence is calculated by a feedforward network which is represented as ${F_{AL}\in R}^{N\times S\times C\times H}$ along the time dimension. As defined in Equation (6), through a multilayer perceptron (*MLP*) module consisting of two-layer 2D CNN, $F_{AL}$ (the output of *MLP*) is the element-wise product of $F_{global}$ to obtain the temporal excitation features ${F_{GLTA}\in R}^{N\times S\times C\times H}$.

(6) $$F_{AL}=\frac{\exp\left({Conv2d\;}_{s}\left({Conv2d\;}_{s}\left(F_{global}\right)\right)\right)}{\sum_{i=1}^{n}\;\exp\left({Conv2d\;}_{s}\left({Conv2d\;}_{s}\left(F_{global}\right)\right)\right)}.$$

#### 2.2.3. Adaptive Temporal Feature Aggregation (ATFA)

**Figure 4:**
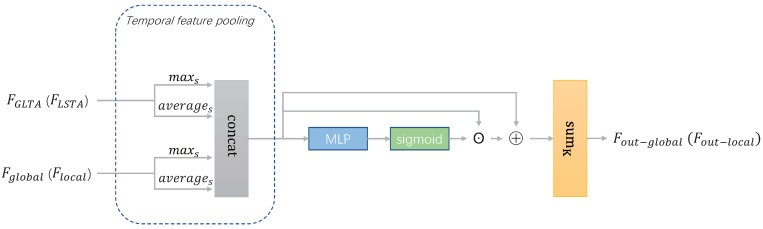
The overview of ATFA.

In this module, as shown in Figure 4, an adaptive temporal feature aggregation is proposed. First, the max pooling and the average pooling are applied to reduce the dimension of the input features in the temporal dimension *S*, and concatenate them. The max pooling can represent the salient information of the sequence, while the average pooling can represent the overall information of the sequence. The temporal feature pooling can be formulated as Equations (7) and (8) to obtain the features $F_{cat-global}{\in R}^{N\times K\times C\times H}$ and $F_{cat-local}{\in R}^{N\times K\times C\times H}$ from $F_{global}$, $F_{GLTA}$ and $F_{local}$, $F_{LSTA}$, where *K* denotes the number of features after the temporal feature pooling.

(7) $$F_{cat-global}\;=\;cat\left({max}_{s}\left(F_{global}\right),{average}_{s}\left(F_{global}\right),{max}_{s}\left(F_{GLTA}\right),{average}_{s}\left(F_{GLTA}\right)\right),$$

(8) $$F_{cat-local}\;=\;cat\left({max}_{s}\left(F_{local}\right),{average}_{s}\left(F_{local}\right),{max}_{s}\left(F_{LSTA}\right),{average}_{s}\left(F_{LSTA}\right)\right).$$

Then, in order to adaptively select the feature representations among them and enhance the discriminative power of selected features, multilayer perceptronis introduced to score the splicing dimension of $F_{cat}$ and perform a weighted summation over the splicing dimension *K*. This process can be represented by Equations (9) and (10) to obtain the output features $F_{out-global}{\in R}^{N\times C\times H}$ and $F_{out-global}{\in R}^{N\times C\times H}$, respectively.

(9) $$\left.F_{out-global}=\sum_{i=1}^{k}\;\left(Sigmoid\right.\left(MLP\left(F_{cat-global}\right)\right)•F_{cat-global}+\;F_{cat-global}\right),$$

(10) $$\left.F_{out-local}=\sum_{i=1}^{k}\;\left(Sigmoid\right.\left(MLP\left(F_{cat-local}\right)\right)•F_{cat-local}+\;F_{cat-local}\right).$$

### 2.3. Personal Physiological Feature Module

#### 2.3.1. Estimating Human Physiological Information (HPI)

**Figure 5:**
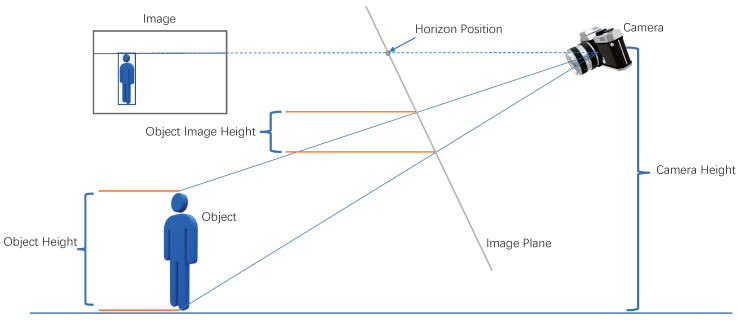
The real height and width of a person can be estimated from his bounding box in the image as long as the camera viewpoint and setting are known. Detailed illustration and explanation can be found in [22].

As shown in Figure 5, the real height and width of a person and his depth to the camera could be expressed as ($H_{p},W_{p},Z_{p}$); the optical focus length of a monocular camera is defined as *f*; its optical center is defined as ($O_{x},O_{y}$); $\theta_{cam}$ and $\phi_{cam}$ mean the tilt and rotation of the camera; the camera height is expressed as $H_{cam}$. In this research, this paper can estimate the height and width ($H_{img},W_{img}$) of a person in the image by YOLOV5 [21]. Then, the real personal feature information could be described by the camera parameters and image information as follows:

(11) $$Person\left(H_{p},W_{p},Z_{p}\right)=M\left(H_{img},W_{img},H_{cam},f,O_{x},O_{y},\theta_{cam},\phi_{img}\right),$$

As described in [22], zero rolling (or the fact thatthe image has been rotated to account for roll) is assumed to calculate ($H_{p},W_{p},Z_{p}$) as follows:

(12) $$\left\{\begin{array}{cc} Z_{p}= & \frac{f*H_{cam}}{f*sin\left(\theta_{img}\right)-\left(O_{y}-H_{img}\right)*cos\left(\theta_{img}\right))};\\ H_{p}= & \frac{H_{img}*Z_{p}}{f};\\ W_{p}= & \frac{W_{img}*Z_{p}}{f}. \end{array}\right.$$

The readers are referred to more detailed discussion and description of Equation (12) in [22].

**Figure 6:**
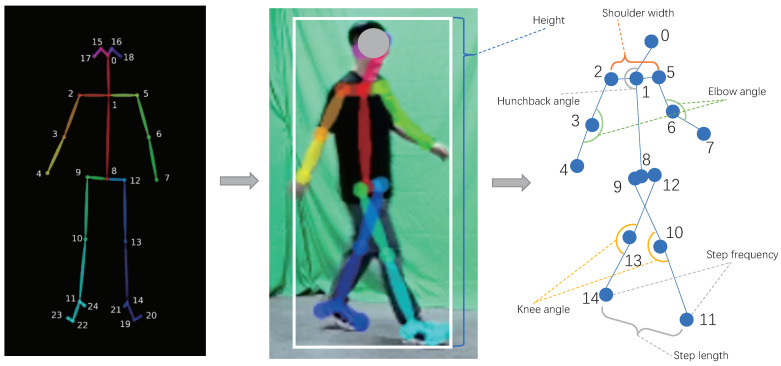
After obtaining the real human height from Equation (12), with the skeleton points extracted from OpenPose [23], this paper can obtain 7 real human physiological information parameters: height, shoulder width, hunchback angle, elbow angle, knee angle, step length, and step frequency.

According to Equation (12), since there is a linear relation between the bounding ($H_{img},W_{img}$) and real human height and width ($H_{p},W_{p}$), as shown in Figure 6, according the skeletal points estimated by OpenPose [23] and the ($H_{img},W_{img}$) obtained from YOLOV5, it is easy to estimate the following seven real pieces of human physiological information: height, shoulder width, hunchback angle, elbow angle, knee angle, step length, and step frequency.

#### 2.3.2. Physiological Feature Extraction (PFE) Module

**Figure 7:**
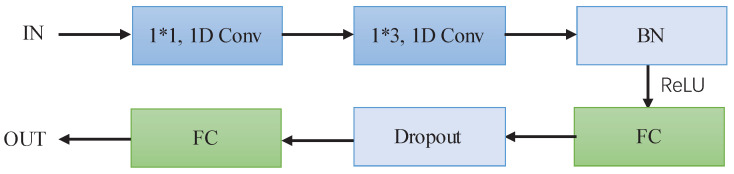
Implementation process of the physiological feature extraction module.

After obtaining each piece of physiological information, in order to enhance the discriminative ability and correlation among these pieces of information, this paper proposes a new network, shown in Figure 7, for physiological feature extraction, where a 1D CNN of size 3 is used to obtain the correlation between each piece of physiological information. After two such 1D CNN layers, a batch normalization (BN) process is adopted to accelerate the convergence of the proposed network, and the output of the BN is applied to a full connection (FC) layer. Then, a dropout layer is selected to avoid the overfitting problem.

Assuming that the feature input is defined as $P_{in}{\in R}^{N\times C\times L}$, where *N* is the batch number, *C* denotes the number of channels and *L* denotes the feature length. This process can be defined as Equation (13), through which the feature output $F_{PFE}{\in R}^{N\times L^{{}^{\prime }}}$ is obtained. Here, $L^{{}^{\prime }}$ is the feature length after passing through the full connection layer.

(13) $$F_{PFE}=FC\left(Conv1d\_3\left(P_{in}\right)\right).$$

### 2.4. Loss Function

As shown in Figure 1, in this work, in order to make the silhouette gait features more distinguishable, the batch all triple loss [24] and cross-entropy loss functions are selected, where the triple loss could increase the compactness within a class and the cross-entropy loss can measure the separability between global classes.

The combined loss function is defined as Equation (14). Within each batch, the triple loss over all samples is defined as Equation (15), where $all\_d_{a,p}$ is the average distance between each anchor and all positive samples, $all\_d_{a,n}$ is the average distance between each anchor and all negative samples, and α is the margin value.

(14) $$L_{\mathrm{combined}}=L_{\mathrm{all}}+\;L_{Cross\;entropy},$$

(15) $$L_{\mathrm{all}}=max\left(all\_d_{a,p}-all\_d_{a,n}+\alpha \;,\;0\right),$$

The triple loss of PFE is also measured by Equation (15). The loss vectors of the silhouette-based module and the PFE module are concatenated to create a new feature vector, and the ID reranking process is performed by measuring the length of this new feature vector.

## 3. Results

To ensure the effectiveness and efficiency of the proposed algorithm, three experiments were carried out: (1) In Section 3.2, we examined the effectiveness of the human physiological information estimation module; (2) in Section 3.3.1, we conducted comparative experiments among the conventional gait recognition algorithms [5,6,25,26] and the proposed method (without HPI and PFE modules) on CASIA-B [20]; (3) we conducted comparative experiments among the proposed method (with HPI and PFE modules) and the baseline methods on the Multi-state Gait dataset, where the real human physiological information could be estimated.

### 3.1. Datasets and Training Details

CASIA-B [20]: There are a total of 124 persons included in this dataset, where each person contains 11 views and each view contains 10 sequences under three walking conditions: normal (NM), carrying a bag or backpack (BG), and wearing coats or jackets (CL). The first six sequences are obtained under NM condition, and the other two sequences are captured with BG and the last two sequences under CL conditions. This paper follows the popular protocol carried out in [8]: the first 74 persons are used for training and the remaining 50 ones for testing. During the test, the first 4=four sequences of NM (NM#1–4) are used as gallery, the remaining six sequences (regard as probe) are divided into three subsets according to the walking conditions: the NM subset contains NM#5–6, the BG subset contains BG#1–2, and the CL subset contains CL#1–2.

Multi-state Gait dataset (the approved informed consent was obtained from all the subjects in this dataset. Their personal images were authorized to be used for the academic research): Since the setting and parameters of cameras are not included in the CASIA-B dataset, the new Multi-state Gait dataset was created, where the camera parameters and settings were recorded, through which the HPI module could estimate the real human physiological information.

**Figure 8:**
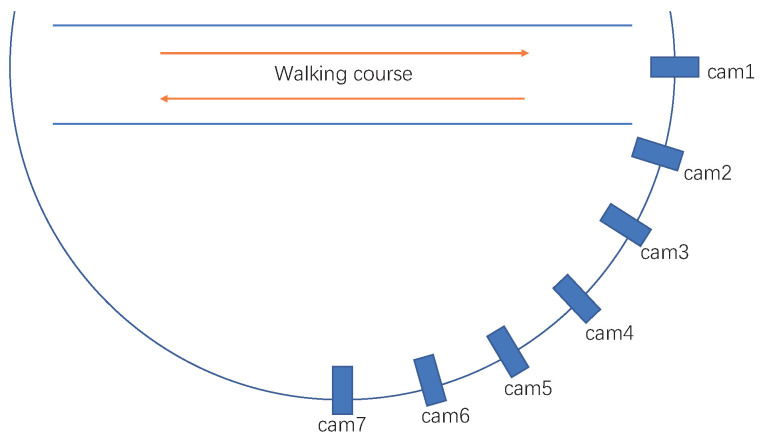
The setup of cameras in collecting data for the Multi-state dataset. There are 7 Hikvision cameras applied in this scene and the interval between two adjacent cameras is 15 degrees. The subject person was instructed to walk forward and backward along the same route.

**Figure 9:**
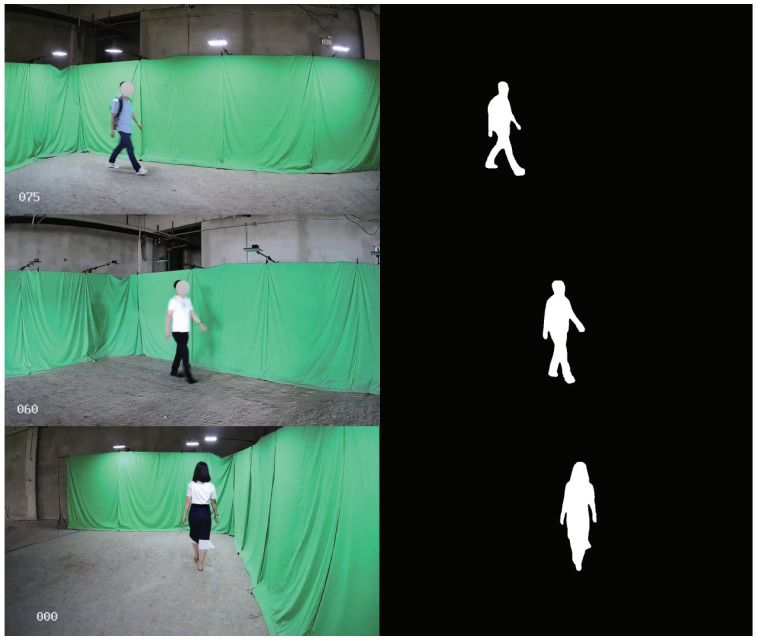
Illustration of the proposed Multi-state Gait dataset. The gait silhouette map was extracted by using the Mask R-CNN [26].

As shown in Figure 8, all the data in the Multi-state Gait dataset were captured by seven Hikvision cameras (the interval between two adjacent cameras is 15 degrees) at 20 fps with the resolution of 1280 × 720 pixels. There were 60 subject persons included in this dataset, and each person was instructed to walk in bio-directions (forward and backward). Therefore, the viewing angles varied from 0–90${}^{\circ }$ and 180–270${}^{\circ }$, respectively. All the cameras were set at 2 m height from the ground with their pitch angles fixed as 5${}^{\circ }$. With the help of OpenCV, all the data were collected by a desktop PC with the AMD R9 5950X CPU, 32 GB memory, and NVIDIA RTX3090. During the training and test process, the software condition is Pytorch1.8 + Cuda10.1 + Pycharm + Ubuntu. Figure 9, shows some collected samples, and the gait silhouette sequences were extracted by using the Mask R-CNN [26].

Similar to [27], this dataset contains 60 persons, where each person contains 14 angles (0, 15, 30, 45, 60, 75, 90, 180, 195, 210, 225, 240, 255, 270 degrees) and each angle contain 14 sequences: six sequences for NM, four sequences of BG, and four sequences for CL. Here, the first 34 persons are selected for training and the remaining 26 persons for testing. During the test, the first four sequences of NM (NM#1–4) are used as gallery, and the remaining 10 sequences (regard as probe) are divided into three subsets: the NM subset contains NM#5–6, the BG subsets contains BG#1–4, and the CL subset contains CL#1–4.

Training Details in CASIA-B: The gait silhouette map inputted into the network is set to 64 × 44 pixels, and the images are aligned according to the method of [27]. Each gait cycle contains 30 frames from each view angle (a total of 11 angles). The margin in the triple loss $L_{all}$ is set to 0.2, the Adam optimizer is applied in the training process, and the learning rate is set to 1 × 10${}^{-4}$. After 120K iterations, the learning rate is adjusted to 1 × 10${}^{-5}$, and the local optimization for the proposed network was achieved after 5K iterations.

Training Details in the Multi-State Gait Dataset: The resolution of the gait silhouette map inputted into the network is 64 × 44 pixels, and all the images are aligned according to the method of [27]. Each gait cycle contains 30 frames from each view angle (a total of 11 angles). During training, the Adam optimizer is used, the margin in the triple loss $L_{all}$ is set to 0.2, and the learning rate is set to 1 × 10${}^{-4}$. Because this dataset is small, a total of 70Kiterations are performed. Since both GaitSet [5] and GaitPart [6] were selected as the comparative baseline methods, they were also trained in this dataset. GaitSet [5] and GaitPart [6] use the same parameter settings to perform 70K iterations, respectively.

Regarding the PFE module in the proposed work, the size of a single piece of information inputted to the network is set as 1 × 7, the dropout layer parameter is set to 0.5, in which the margin in the triple loss $L_{hard}$ is set to 0.4, the Adam optimizer is also used, and the learning rate is set to 1 × 10${}^{-4}$ for 30 iterations.

For either dataset, the training process of the proposed model is implemented in Pytorch1.8 + Cuda10.1 by using one NVIDIA RTX3090 GPU under the Ubuntu conditions.

### 3.2. Efficiency Evaluation of Physiological Information Computing

In order to verify the effectiveness of the HPI module in the proposed work, this paper selected four persons under three angles from the Multi-state Gait dataset. Here, two different experiments were performed: the static measurement for angle evaluation (elbow, knee, and hunchback angles) from static images, and the dynamic measurement for length evaluation (height, shoulder width, step length, and step frequency) from video sequences.

Table 1 shows the detailed experimental results of the static estimation error evaluation of the HPI module, under the three angles. The error rate is the ratio between $\;A_{EST}-A_{GT}∥$ and $∥A_{GT}∥$. Here, $A_{EST}$ means the estimated angle by the HPI module and $A_{GT}$ represents the ground truth angle. In total, the estimation error of the HPI module for elbow and knee angles is around 6% and the hunchback angle has more estimation error (up 9.4%). This is because of the unstable skeleton point 0 (shown in Figure 6) due to the variation of view angles. Another important element lies in the experimental error that was caused by the displacement between the skeleton points estimated by OpenPose [23] and the position in which the real medical instrument was placed.

**Figure 10:**
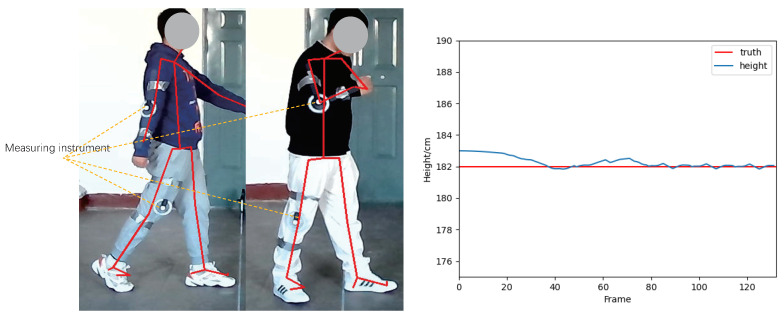
HPI evaluation. Left image: the ground truth value of $A_{GT}$ is obtained through the medical instrument that is fixed on the test part of the human. Right image: Kalman filter is applied during the dynamic measurement experiment to compress the effect of random noise data.

Table 2 shows the detailed information in the dynamic measurement experiment. Here, the target persons were required to walk from different angles. Regarding the step length measurement, the ground truth value was collected by measuring the distance between two footprints of a person, where his/her shoe’s bottom was painted with ink. The ground truth (step length) was the mean value of all manually measured step lengths during a walking sequence. As shown in the right image of Figure 10, a Kalman filter is applied to the estimated HPI features (such as height, shoulder width, etc.) to eliminate the effect of random noise. The estimation error of each HPI varies from 1.2% to 8.1%; this is because the real person’s height is estimated from the detection result of YOLOV5 [21], where the bounding box of the target person is quite accurate. While the shoulder width, step length, and step frequency were estimated from the skeleton points from OpenPose [23], the positions of skeleton points become unstable due to the motion blur in the test images; the estimation error of such information is higher than that of the person’s real height.

### 3.3. Comparison with State-of-the-Art Methods

#### 3.3.1. Comparative Experiments on CASIA-B Dataset

To confirm the effectiveness of the proposed method, the comparative experiment was performed on the CASIA-B dataset among the proposed method and the other four methods: GaitSet [5], GaitPart [6], CNN-LB [8], and GaitNet [25]. Here, as shown in Table 3, 50 persons were selected as the target persons and the detailed training information could be found in the aforementioned section. All the target persons contained 11 view angles under three conditions: normal walking (NM), carrying a bag or backpack (BG), and wearing coats or jackets (CL). It is obvious that the proposed method achieved superior performance to the other methods over most view angles under all conditions (rank-1 under NM, BG, and CL conditions). The mean value of the recognition accuracy in the proposed method was 96.5% under NM, 92.6% under BG, and 79.8% under CL. Such stable ranking over all 11 view angles under three conditions could prove the effectiveness of the proposed methods. The superior performance of the proposed method lies in the fact that, compared with the other works such as GaitPart (extracting features from three or a fixed number of frames), more effective gait features are extracted by the LSTA and GLTA modules, where both the local three continuous frames and the whole gait cycle are processed. This is because the movement frequency of different people may change greatly, and extracting the gait feature at a fixed image interval may not produce enough information for recognition, while the proposed method can obtain more useful feature from the whole gait cycle. Therefore, it is natural that the more powerful gait features (obtained by LSTA and GLTA) could help to improve the gait recognition accuracy.

#### 3.3.2. Comparative Experiment on the Multi-State Gait Dataset

Since the CASIA-B [20] dataset does not contain the necessary information such as camera setting and internal parameters, which is required by the HPI module, this paper performed another comparative experiment on the Multi-state Gait dataset to confirm the effectiveness of the proposed method. Here, GaitSet [5] and GaitPart [6] are selected as the compared baseline methods due to their good performance on the CASIA-B dataset (shown in Table 3). Here, 26 persons are selected as the tested targets from 14 view angles under three conditions (NM, BG, and CL). In Table 4, “ours_1” means the LSTA+GLTA+ATFA in the proposed work, while “ours_2” represents the method containing all the proposed modules (LSTA+GLTA+ATFA+HPI+PFE). Among all the 14 view angles, “ours_2” ranked number 1 for nine angles under NM and BG conditions and ranked number 1 for 10 angles under the CL condition. From all 14 angles under three conditions, the average recognition accuracy of “ours_2” is superior to all the compared methods, which implies that introducing the HPI information with the PFE module could efficiently improve the performance in gait recognition tasks. This is because, compared with the silhouette images which may become completely different due to the variation of camera viewing angles, a person’s real physiological information could hardly change (despite different viewing angles). Therefore, it is not strange that introducing such stable personal gait features will improve the performance of a gait recognition method. Experiments in the following section prove that such an idea is also suitable for the other compared methods.

In addition, the FLOPs of the proposed model and compared baseline works are also calculated to measure their computational complexity. Under the same data input, the FLOPs of the proposed model (145 M) are in between GaitSet [5] and GaitPart [6], which is 20% lower than GaitSet (183 M) and 37% higher than GaitPart (106 M). Since GaitSet contains a more complex network structure (including feature pyramid structure) than the proposed work, it is reasonable that the computation cost of the proposed method is less than that of GaitSet. Compared with GaitPart, besides the similar network to extract features for short-term, the proposed work contains more complex structures, such as GLTA, ATFA, and PFE, to compute the long-term gait feature and real human physiological feature. Therefore, the proposed method could achieve better recognition accuracy at the cost of more computational complexity than GaitPart.

### 3.4. Ablation Study

Besides the overall performance evaluation, the effectiveness of each module in the proposed methods were also investigated.

Firstly, the validity of each module of the gait silhouette part on the CASIA-B [20] dataset was verified. As shown in Table 5, the baseline of the work is that the LSTA module and the GLTA and ATFA modules are also selected to verify how to combine them to improve the performance of the method. Through these experiments, directly applying the LSTA module for gait recognition will lead to a similar result to the well-known GaitPart [6], while the combination of LSTA and GLTA could improve the performance under all conditions and the mean recognition accuracy could reach 89.2%, and by combining LSTA with GLTA and ATFA, the performance of the proposed work is further improved to 89.6% in the mean value of recognition rate. The improvements caused by introducing LSTA and ATFA modules are, respectively, 0.8% and 0.4%, which indicates that, compared with the adaptive adjustment of feature weights (by ATFA), the global gait feature could be more useful to improve the performance of the proposed method.

Since the effectiveness of HPI and PFE cannot be verified in CASIA-B [20], the Multi state Gait dataset was applied to examine these two modules. In Table 6, the performance of HPI and PFE modules in improving the gait recognition accuracy were investigated. Here, “Baseline” means the LSTA + GLTA + ATFA modules in the proposed method, “Baseline + HPI” represents directly applying the obtained real human physiological information in the silhouette-based network, and “Baseline + HPI + PFE” denotes the combination of the baseline method with the human physiological features extracted through the PFE network. The performance of the baseline method is quite similar to that of the well-known GaitPart [6] work, and it is obvious that directly applying the HPI information can only slightly improve the gait recognition accuracy. Through this experiment, the real human physiological features obtained through the HPI+PFE can achieve the best improvement in gait recognition.

### 3.5. Transplantation Study

Besides the proposed method, as shown in Table 7, the authors also investigated whether the HPI and PFE modules could help to increase the accuracy of other gait recognition methods or not. Here, according to the network structure of GaitPart [6], a weight parameter γ is introduced to the real personal physiological features, so that the ratio of the gait silhouette feature length to the real personal physiological feature length is set to 32:1. It is interesting to see that, with the help of the HPI and PFE module proposed in this paper, the average recognition accuracies of GaitSet [5] and GaitPart [6] increased by0.53% and 0.47%, respectively. This can be considered as proof of the idea that the unique real human physiological information can be helpful to improve the performance of a gait recognition algorithm.

## 4. Discussion

In the future, several improvements should be considered:

(1) Experiments on the other large public datasets (such as the OUMVLP Dataset) should be performed. Due to the limitations of hardware, the authors cannot test the proposed method on such large datasets. It is believed that such experiments could be achieved with more powerful GPU hardware.
(2) Determining how to extract more accurate HPI features should be investigated. Currently, since only the monocular images were applied, the skeleton points of a person may be invisible due to variation of view angles. The 3D skeleton points are considered be a solution to this problem, and such points could be obtained through the RGB-D camera, stereo vision, or other 2D–3D neural networks through successive frames.
(3) More clear test images should be applied in future research. As the motion blur has caused many experimental errors in the work (because the estimation of skeleton points becomes unstable), the proposed method should be applied to the test images obtained through high-speed cameras rather than the normal ones (such as the shutter speed of 20 fps).

## 5. Conclusions

In this paper, a new gait recognition method was brought about, which is based on the fusion of gait silhouette features and real personal physiological features. To deal with the variation of gait frequency among different people, both the short-term (three frames) and long-term (whole gait cycle) gait features are extracted by the novel LSTA and GLTA modules for improving the recognition accuracy. As for the appearance variation of silhouette images under different viewing angles, the real human physiological information calculated from monocular images is selected so as to provide more robust gait features. The final gait recognition is achieved by reranking among the feature vectors concatenated by the features obtained from LSTA, GLTA, and human physiological information. The effectiveness and efficiency of the proposed method was proved through the massive comparative experiments among the proposed methods and the other well-known algorithms on both the public dataset and the newly brought about Multi-state one. Since the proposed method is mainly designed for intelligent security monitoring systems, its performance will depend on several things such as the image resolution, camera capture speed, etc. This is because low image resolution will lead to more estimation error for the skeleton points and low capture speed will cause motion blur, which will not only affect the estimation of skeleton points but also the quality of the silhouette image. One of the future work directions in our research is to introduce the high-speed camera as well as carry out the experiment under more real-life scenes.

## Figures and Tables

**Table 1:** Static measurement error of HPI with 4 persons under 3 different angles.

Person	Estimation Error								
	30${}^{\circ }$	45${}^{\circ }$	60${}^{\circ }$	30${}^{\circ }$	45${}^{\circ }$	60${}^{\circ }$	30${}^{\circ }$	45${}^{\circ }$	60${}^{\circ }$
	Elbow Angle			Knee Angle			Hunchback Angle		
ID1	6.6%	4.6%	4.7%	5.8%	5.4%	6.1%	10.5%	9.7%	11.2%
ID2	8.6%	4.8%	10.7%	8.4%	6.5%	9.3%	7.7%	6.7%	9.6%
ID3	5.6%	5.9%	5.9%	6.3%	3.8%	3.3%	12.7%	8.2%	8.7%
ID4	4.4%	6.7%	6.4%	4.5%	6.5%	5.5%	8.6%	9.8%	8.9%
Mean	6.2%			6.0%			9.4%		

**Table 2:** Dynamic measurement error of physiological information.

Person	Estimation Error											
	30${}^{\circ }$	45${}^{\circ }$	60${}^{\circ }$	30${}^{\circ }$	45${}^{\circ }$	60${}^{\circ }$	30${}^{\circ }$	45${}^{\circ }$	60${}^{\circ }$	30${}^{\circ }$	45${}^{\circ }$	60${}^{\circ }$
	Height			Shoulder Width			Step Length			Step Frequency		
ID1	1.1%	0.9%	1.0%	5.1%	2.5%	7.6%	9.4%	8.8%	8.2%	6.5%	2.3%	5.8%
ID2	2.1%	1.1%	0.5%	1.1%	12.1%	7.4%	7.5%	8.2%	7.7%	4.3%	6.6%	6.6%
ID3	0.9%	2.8%	2.2%	0.8%	2.8%	5.7%	10.2%	5.5%	7.5%	6.3%	6.7%	5.9%
ID4	0.7%	0.9%	0.3%	2.3%	3.3%	5.5%	7.7%	7.9%	8.4%	2.2%	5.2%	4.3%
Mean	1.2%			4.7%			8.1%			5.2%		

**Table 3:** Comparative experimental results in the CASIA-B [20] dataset among the proposed method and other methods, excluding identical-view cases. The bolds indicate the best accuracy in this state.

Gallery NM#1–4		0–180${}^{\circ }$											Mean
**Probe**		**0**	**18**	**36**	**54**	**72**	**90**	**108**	**126**	**144**	**162**	**180**	
NM #5–6	CNN-LB [8]	82.6	90.3	96.1	94.3	90.1	87.4	89.9	94.0	94.7	91.3	78.5	89.9
	GaitSet [5]	90.8	97.9	**99.4**	96.9	93.6	91.7	95.0	97.8	98.9	96.8	85.8	95.0
	GaitNet [25]	91.2	92.0	90.5	95.6	86.9	92.6	93.5	96.0	90.9	88.8	89.0	91.6
	GaitPart [6]	**94.1**	**98.6**	99.3	**98.5**	94.0	92.3	95.9	**98.4**	99.2	97.8	90.4	96.2
	ours	93.4	98.4	99.3	98.4	**95.1**	**93.2**	**96.4**	98.3	**99.4**	**97.9**	**92.2**	**96.5**
BG #1–2	CNN-LB [8]	64.2	80.6	82.7	76.9	64.8	63.1	68.0	76.9	82.2	75.4	61.3	72.4
	GaitSet [5]	83.8	91.2	91.8	88.8	83.3	81.0	84.1	90.0	92.2	**94.4**	79.0	87.2
	GaitNet [25]	83.0	87.8	88.3	93.3	82.6	74.8	89.5	91.0	86.1	81.2	85.6	85.7
	GaitPart [6]	89.1	94.8	96.7	**95.1**	88.3	84.9	89.0	93.5	96.1	93.8	85.8	91.5
	ours	**90.1**	**96.1**	**97.0**	95.0	**90.6**	**85.4**	**90.7**	**94.8**	**97.5**	94.3	**87.1**	**92.6**
CL #1–2	CNN-LB [8]	37.7	57.2	66.6	61.1	55.2	54.6	55.2	59.1	58.9	48.8	39.4	54.0
	GaitSet [5]	61.4	75.4	80.7	77.3	72.1	70.1	71.5	73.5	73.5	68.4	50.0	70.4
	GaitNet [25]	42.1	58.2	65.1	70.7	68.0	70.6	65.3	69.4	51.5	50.1	36.6	58.9
	GaitPart [6]	70.7	**85.5**	**86.9**	83.3	77.1	72.5	76.9	82.2	83.8	80.2	66.5	78.7
	ours	**71.2**	84.4	86.7	**83.3**	**79.6**	**76.6**	**79.3**	**83.8**	**85.0**	**80.9**	**67.2**	**79.8**

**Table 4:** Comparison results in the Multi-state Gait dataset, excluding identical-view cases. Here, our_1 does not include real personal physiological feature, and ours_2 includes gait silhouette features and real personal physiological features. The bolds indicate the best accuracy in this state.

Gallery NM #1–4		0–270${}^{\circ }$														
**Probe**	**Model**	**0**	**15**	**30**	**45**	**60**	**75**	**90**	**180**	**195**	**210**	**225**	**240**	**255**	**270**	**MEAN**
NM #5–6	GaitSet [5]	79.7	87.4	91.8	96.2	91.8	76.9	77.5	**79.7**	87.9	94.0	92.9	91.2	81.3	76.9	86.1
	GaitPart [6]	76.4	89.0	96.7	96.7	95.1	87.9	86.8	76.9	91.8	**97.8**	**99.5**	**99.5**	90.7	**87.9**	90.9
	ours_1	81.9	91.2	96.7	96.2	95.6	87.9	87.4	75.8	92.9	95.1	97.8	97.3	90.7	84.6	90.7
	ours_2	**81.9**	**91.2**	**96.7**	**96.7**	**95.6**	**88.5**	**87.9**	75.8	**92.9**	95.1	97.8	97.3	**90.7**	85.7	**91.0**
BG #1–4	GaitSet [5]	**73.9**	81.2	89.3	91.5	87.1	71.4	75.8	**78.3**	83.2	90.1	90.7	85.7	75.8	80.0	82.4
	GaitPart [6]	69.5	84.3	91.8	**94.8**	86.8	78.0	78.9	66.2	81.2	92.6	93.1	**89.6**	**86.8**	79.4	83.8
	ours_1	70.6	85.5	92.6	93.1	91.2	81.0	81.6	72.8	88.0	92.3	92.3	87.1	83.5	81.9	85.3
	ours_2	70.6	**86.3**	**93.7**	93.7	**91.2**	**82.1**	**81.6**	73.0	**88.9**	**93.4**	**92.6**	88.3	84.9	**82.1**	**85.9**
CL #1–4	GaitSet [5]	67.6	76.9	76.4	75.8	72.5	67.6	60.4	62.1	70.9	74.2	74.2	74.7	55.0	61.0	69.2
	GaitPart [6]	73.0	72.4	82.3	84.0	**80.1**	71.9	**74.1**	**67.5**	70.2	81.8	75.7	64.2	58.7	**64.7**	72.9
	ours_1	73.6	78.0	87.9	85.2	75.8	70.9	68.7	63.7	76.9	83.0	80.2	74.7	61.5	63.2	74.5
	ours_2	**73.7**	**78.6**	**87.9**	**86.3**	76.9	**72.5**	69.2	63.8	**78.0**	**84.1**	**80.8**	**75.3**	**63.2**	63.7	**75.3**

**Table 5:** Ablation experiments on CASIA-B [20] (50 persons, 11 view angles under 3 conditions), excluding identical-view cases. The bolds indicate the best accuracy in this state.

Model	Rank-1%			
	NM	BG	CL	Mean
GaitSet [5]	95.0	87.2	70.4	84.2
GaitPart [6]	96.2	91.5	78.7	88.8
our				
Baseline (LSTA)	96.4	91.4	77.4	88.4
Baseline (LSTA) + GLTA	**96.7**	91.8	79.2	89.2
Baseline (LSTA) + GLTA + ATFA	96.5	**92.6**	**79.8**	**89.6**

**Table 6:** For real personal physiological features, we conducted ablation experiments in the Multi-state Gait dataset, excluding identical-view cases. The bolds indicate the best accuracy in this state.

Model	Rank-1%			
	NM	BG	CL	Mean
GaitSet [5]	86.07	82.43	69.23	79.24
GaitPart [6]	90.89	83.78	72.90	82.52
our				
Baseline	90.74	85.25	74.53	83.51
Baseline + HPI	90.81	85.34	74.64	83.60
Baseline + HPI + PFE	**90.97**	**85.89**	**75.28**	**84.05**

**Table 7:** Translating the HPI and PFE modules to other methods in the Multi-state Gait dataset. The bolds indicate the best accuracy in this state.

Model	Rank-1%			
	NM	BG	CL	Mean
GaitSet [5]	86.07	82.43	69.23	79.24
GaitSet [5] + HPI + PFE	**86.34**	**82.75**	**70.21**	**79.77**
GaitPart [6]	90.89	83.78	72.90	82.52
GaitPart [6] + HPI + PFE	**91.05**	**84.38**	**73.61**	**83.01**

## Data Availability

Not applicable.
